# Bradycardia caused by interaction of venlafaxine and cyclosporine: A case report

**DOI:** 10.22088/cjim.10.4.463

**Published:** 2019

**Authors:** Marzieh Azizi, Forouzan Elyasi, Fatemeh Niksolat Roodposhti

**Affiliations:** 1 Sexual and Reproductive Health Research Center, Nasibeh Nursing and Midwifery Faculty, Mazandaran University of Medical Sciences, Sari, Iran; 2 Department of Psychiatry, Psychiatry and Behavioral Sciences Research Center and addiction Institute, School of Medicine, Mazandaran University of Medical Sciences, Sari, Iran; 3 Department of Internal Medicine, Orthopedic Research Center, Mazandaran University of Medical Sciences, Sari, Iran

**Keywords:** Bradycardia, Venlafaxine, Cyclosporine, Serotonin-Norepinephrine reuptake inhibitors, Selective serotonin reuptake inhibitors.

## Abstract

**Background:**

Selective serotonin reuptake inhibitors (SSRIs) and serotonin-norepinephrine reuptake inhibitors (SNRIs) are prescribed widely for the treatment of depression, anxiety disorders and other psychiatric disorders. Although antidepressants are considered as a safety drug category but unexpected cardiovascular events have been reported as the most serious complications. The aim of this study was to introduce a case presentation on bradycardia due to the drug interference of venlafaxine and cyclosporine.

**Case presentation:**

The patient was a 38-year old woman diagnosed with systemic lupus erythematosus 5 years ago, who was admitted to a general educational hospital in northern Iran due to intensified rheumatologic symptoms and complaining about abdominal pain. Cyclosporine tab were administered to the patient, 50 mg twice daily. Two weeks after the administration of cyclosporine, the level of blood cyclosporine was checked. The patient became bradycardic after starting a single dose of venlafaxine (heart rate 52 ppm). Cardiac assessment showed no reason for bradycardia and it subsided after a drop of venlafaxine.

**Conclusion:**

As a result of the potential adverse drug interactions between cyclosporine and antidepressants such as venlafaxine, physicians should be aware of the possibility of bradycardia in the simultaneous prescription of these drugs in cases.

Selective serotonin reuptake inhibitors (SSRIs) and serotonin-norepinephrine reuptake inhibitors (SNRIs) are prescribed widely for the treatment of depression, anxiety disorders and other psychiatric disorders (1). The cause of the widespread use of SSRIs and SNRIs drugs include relative safety, appropriate tolerability and low toxicity (2). Although antidepressants are considered as safety drugs but unexpected cardiovascular events such as arrhythmia or sudden heart arrest or cardiac death even in an individual with no history of cardiac problems have been reported as the most serious complications of these drugs (3, 4). SNRIs elevated norepinephrine and serotonin levels, can accelerate the activity of the cardiac sympathetic system and lead to a mild increase in heart rate and systemic blood pressure. Epidemiological studies showed that blood pressure monitoring is proposed for patients receiving SNRIs, especially venlafaxine. This drug with blocking sodium channels may show QTc wave prolongation in electrocardiogram results (5, 6). Even though tachycardia is more common in patients with venlafaxine, some studies showed bradycardia, as a result of venlafaxine consumption (7, 8). A case report has been reported on drug interactions resulting from the simultaneous consumption of cyclosporine and antidepressants such as nefazodone and venlafaxine which led to an increase in serum creatinine levels and increased levels of cyclosporine metabolism in the blood. Both nefazodone and venlafaxine led to inhibitors of CYP3A3 /4 isoenzyme (9). 

Given the awareness of the inhibitory effects of SNRIs agents on hepatic cytochrome isoenzymes, it may predict the probability of a potential drug interaction between SNRIs and cyclosporine. The aim of this study was to introduce a case presentation on bradycardia due to the drug interference of venlafaxine and cyclosporine.

## Case presentation

The patient was a 38 year old woman, married, third grade middle school, unemployed, diagnosed with systemic lupus erythematosus 5 years ago, and was admitted to a general educational hospital in northern Iran due to chronic rheumatologic symptoms and complaining about abdominal pain. The patient mentioned that this pain was not related to eating and defecation and was not exacerbated by her activities. The results of initial laboratory tests and sonography were normal. 

**Figure 1:**
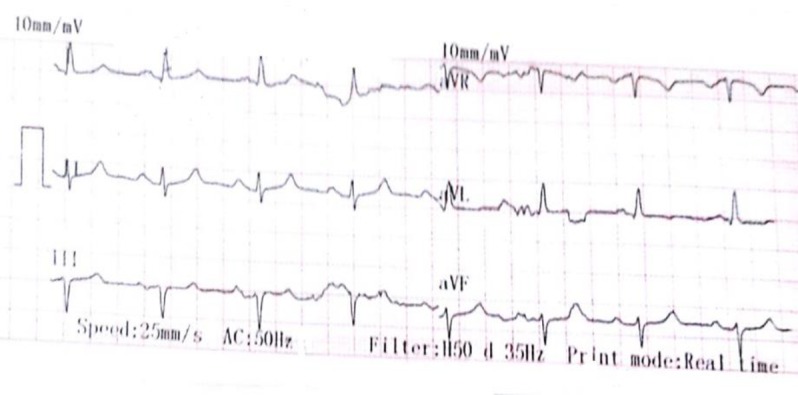
The patient's ECG before prescription of venlafaxine which showed no bradycardia

**Figure 2:**
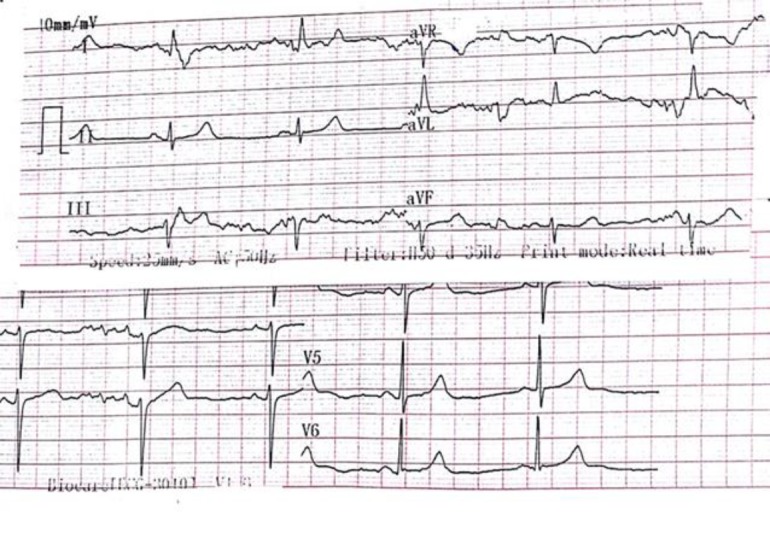
The patient's ECG before prescription of Venlafaxine which showed bradycardia

Cyclosporine tab 50 mg twice daily were administered to the patient. Two weeks after cyclosporine was prescribed, the blood cyclosporine level was checked. She had a history of treatment with sertraline, trazodone, quetiapine and duloxetine due to mood disorders. The patient also mentioned a history of suicidal attempts in the past. At that time, she used quetiapine 100 mg nightly. In the psychiatric examination, she was oriented, alert, had a dysphoric mood and was able to communicate well. The patient mentioned that she had attempted suicide twice due to marital conflicts but her children prevented the final action to suicide. In the period of her hospitalization, she had no suicidal thoughts. Quetiapine continued and venlafaxine was started at 37.5 mg per day. For 2 days, the patient received only cyclosporine, and had no bradycardia (figure 1) but after prescribing a single dose of venlafaxine, the patient became bradycardic (heart rate 52 ppm) (figure 2). In cardiac counseling, a 24-h Holter monitoring was done for the patient and the left ventricular ejection fraction (LVEF) was 55. Also, for assessment of QTc prolongation, daily electrocardiography (ECG) was requested 72 h later. Due to bradycardia, it was advised to discontinue venlafaxine and quetiapine. Also, the rheumatologist discontinued cyclosporine prescription. Psychiatric counseling was requested as a result of continuing psychiatric symptoms and she was visited by the psychiatrist twice a day for 72 h. Within 48 h of discontinuation (venlafaxine and cyclosporine), bradycardia was gradually eliminated.

## Discussion

In this study, the patient showed bradycardia due to venlafaxine. The Naranjo adverse drug reactions (ADR) probability scale is shown in table 1. According to the results of this scale, venlafaxine with a probable ADR (score 8) was considered as the cause of bradycardia in this patient (13).

Venlafaxine is a relatively new and commonly prescribed antidepressant from the SNRIs category and it is mainly approved and prescribed in the case of a major depressive disorder and general anxiety disorder (7). Studies have shown that this drug causes a reuptake of both serotonin and norepinephrine receptors and also has a weak effect on histamine and alpha-adrenergic receptors (14, 15). According to literatures, this drug is a relatively safe agent and patients treated with venlafaxine compared to other drugs such as tricyclic antidepressants (TCAs) showed a lower risk of drug's interactions. Hence, it is considered as an appropriate selection in the elderly or patients with disabilities (16) but cardiac abnormalities such as changing on QTc prolongation because of increased concentrations of venlafaxine has been reported in some cases. These changes can be explained through blocking the fast current of sodium in ventricular myocytes (17). 

It is only contraindicated in patients with an identified risk of a serious cardiac ventricular arrhythmia or with uncontrolled hypertension. Also, it has been shown that compared to other effective antidepressants, venlafaxine is associated with the greatest risk of death from overdose (18). Results about this negative effect on cardiovascular system were controversial such that in a retrospective study, elderly patients who received venlafaxine were not only low to moderate in doses of this drug. Although there were no adverse cardiovascular problems, but they showed a lower risk of heart failure compared to SSRIs drugs such as sertraline (19).

**Table 1:** The Naranjo adverse drug reaction probability scale: To assess the adverse drug reaction

	**Yes**	**No**	**Don’t ** ** Know**	**Score**
1.Are there previous conclusive report on this reaction	✔			+1
2. Did the adverse event occur after the suspected drug was administered?	✔			+2
3. Did the adverse reaction improve when the drug was continued or a specific antagonist was administered?		✔		0
4. Did the adverse reaction reappear when the drug was readministered?			✔	0
5. Are there alternative causes (other than the drug) that could have on their own caused the reaction?		✔		+2
6. Did the reaction reappear when a placebo was given?			✔	0
7. Was the blood detected in the blood (or other fluids) in concentrations known to be toxic?		✔		+1
8. Was the reaction more severe when the dose was increased or less severe when the dose was decreased?	✔			+1
9. Did the patient have a similar reaction to the same or similar drugs in any previous exposure?			✔	0
10. Was the adverse events confirmed by any objective evidence?	✔			+1

Cyclosporine is an immunosuppressive agent which is used as a proper drug for the treatment of many autoimmune diseases (20). In a case report study in which the patient underwent allogeneic hematopoietic stem cell transplantation, cyclosporine 60 mg/kg was administered for two days and after continuous infusion of cyclosporine, 3mg/kg was prescribed with maintained checking of cyclosporine serum between 250-400 ng/dl. On day 23, serum cyclosporine increased to 450 ng/dl, heart rate decreased to 30-40/min and ECG showed sinus bradycardia. This study showed that there was a significant relationship between the dose of cyclosporine used and the occurrence of bradycardia in the patient (21). 

Two probable mechanisms for bradycardia were considered as induced by the use of cyclosporine. In the first proposed mechanism, Cyclosporine may suppress the sinus node automatically and sinus bradycardia was reported in cases with cyclosporine prescription but this abnormality in the sinus node diminished after discontinuation of this drug (22). The second possible mechanism is that cyclosporine may lead to bradycardia by provoking the parasympathetic nervous system but studies have suggested that the second possible mechanism is less likely (21, 23). 

A study which assessed the adverse effect of cyclosporine on baroreflexes through the inhibition of testosterone receptors of cardiac vagal control showed that short term cyclosporine treatment led to decreased plasma testosterone level, impaired baroreflexes function, reduced its sensitivity and overall caused to reflex bradycardia (23). The selection of antidepressants depends on many factors such as the current drug prescribed for the patient, and the probable drugs interactions. Most antidepressants are metabolized in the liver through the isoenzyme P450 cytochrome system. The inhibition of this isoenzyme by drugs affect other drugs which need an isoenzyme for metabolism or transformation (14). 

Among the various isoenzymes of P450, CYP3A3/4, plays the most important role in the metabolism of cyclosporine and inhibition of this system leads to decreased cyclosporine metabolism and increased cyclosporine level which cause toxicity in patients. Venlafaxine and nefazodone have the strongest inhibitory action on the CYP3A3/4 isoenzyme and decreased cyclosporine metabolism (9, 16). In this case report, maybe venlafaxine and cyclosporine drug interaction caused bradycardia but it was not specifically clear that which drugs creates bradycardia. Because patient showed no bradycardia during drug consumption of cyclosporine and after adding a single dose of venlafaxine patient showed bradycardia in her ECG and also bradycardia disappeared after discontinuation of venlafaxine, it is probably attributed to single dose of venlafaxine. Clinical studies have shown that the adverse interactions of these drugs depend on many factors such as the concentration of the isoenzyme inhibitor, amount of participation of active metabolites in the isoenzyme inhibitory activity and the level of toxicity of prescribed drugs (9). As a result of the potential adverse drug interactions between cyclosporine and antidepressants such as venlafaxine, physicians should be aware of the possibility of bradycardia in simultaneous prescription of these drugs in cases. Also, it has been proposed that ECG control should be continued during therapy, especially in patients with cardiovascular abnormalities or disorders. 
